# Phosphorylated STAT3 suppresses microRNA‐19b/1281 to aggravate lung injury in mice with type 2 diabetes mellitus‐associated pulmonary tuberculosis

**DOI:** 10.1111/jcmm.15954

**Published:** 2020-10-22

**Authors:** Xianhua Wang, Yuefu Lin, Ying Liang, Yang Ye, Dong Wang, Aer Tai, Shuimiao Wu, Jian Pan

**Affiliations:** ^1^ Department of Quality Control Center for Disease Control and Prevention of Changji Hui Autonomous Prefecture Changji China; ^2^ Department of Prevention Linyi People's Hospital Linyi China; ^3^ Department of Nutrition and Food Hygiene School of Public Health Xinjiang Medical University Urumqi China; ^4^ Department of Laboratory People's Hospital of Changji Hui Autonomous Prefecture Changji China; ^5^ Department of Tuberculosis Center for Disease Control and Prevention of Changji Hui Autonomous Prefecture Changji China; ^6^ Department of Respiratory Medicine Xinjiang Uygur Autonomous Region Chest Hospital Urumqi China; ^7^ Department of Respiratory and Critical Care Medicine Weinan Central Hospital Weinan China; ^8^ Department of Respiratory and Critical Care Medicine The Third People's Hospital of Xinjiang Uygur Autonomous Region Urumqi China

**Keywords:** inflammation, microRNA‐1281, microRNA‐19b, NAFT5, phosphorylated STAT3, pulmonary tuberculosis, Type 2 diabetes mellitus

## Abstract

Type 2 diabetes mellitus (T2DM) is a risk factor for pulmonary tuberculosis (PTB) and increased mortality. This work focused on the functions of phosphorylated STAT3 in lung injury in mouse with T2DM‐associated PTB and the molecules involved. A mouse model with T2DM‐PTB was induced by administrations of streptozotocin, nicotinamide and mycobacterium tuberculosis (Mtb). A pSTAT3‐specific inhibitor AG‐490 was given into mice and then the lung injury in mice was observed. The molecules involved in AG‐490‐mediated events were screened out. Altered expression of miR‐19b, miR‐1281 and NFAT5 was introduced to identify their involvements and roles in lung injury and PTB severity in the mouse model. Consequently, pSTAT3 expression in mice with T2DM‐associated PTB was increased. Down‐regulation of pSTAT3 by AG‐490 prolonged the lifetime of mice and improved the histopathologic conditions, and inhibited the fibrosis, inflammation, Mtb content and number of apoptotic epithelial cells in mouse lung tissues. pSTAT3 transcriptionally suppressed miR‐19b/1281 expression to up‐regulate NFAT5. Inhibition of miR‐19b/1281 or up‐regulation of NFAT5 blocked the protective roles of AG‐490 in mouse lung tissues. To conclude, this study evidenced that pSTAT3 promotes NFAT5 expression by suppressing miR‐19b/1281 transcription, leading to lung injury aggravation and severity in mice with T2DM‐associated PTB.

## 1 INTRODUCTION

Type 2 diabetes mellitus (T2DM) is a major risk factor for tuberculosis (TB) bringing two to eight folds at risk for active TB development, and the nowadays explosive DM pandemic has posed a health priority regarding DM and TB especially in places where TB infection is rampant.^1^, ^2^, ^3^ TB is a leading cause of death from a single infectious disease agent which infected approximately one‐fourth population in the world with more than 10 million people developing active disease annually, predominately active pulmonary TB (PTB), through the infectious mycobacterium tuberculosis (Mtb).^4^, ^5^, ^6^ The first‐line treatment for drug‐sensitive PTB comprises rifampicin, isoniazid, pyrazinamide and ethambutol for over 6 months treatments.^7^ In addition to susceptibility to Mtb infection, T2DM also imposes greater severity of TB disease by influencing disease presentation and increasing unresponsiveness to TB therapy as well as the risk of failure in treatment, relapse and death.^8^, ^9^ Considering the large coverage and the death incidences, developing new understandings and potential therapeutic options for PTB, especially for T2DM‐associated PTB is of great significance.

The signal transducer and activator of transcription (STAT) family members are transcription factors activated by Janus kinase (JAK) proteins, which play important functions in regulating cell proliferation, differentiation, apoptosis and the inflammatory and immune responses.^10^ Particularly, the STAT3 pathway plays unique roles in inflammatory responses and bacterial infection.^11^ It has also been summarized to participate in the replication and pathogenesis of many different human and animal viruses and lead to detrimental effects of viral infection such as oncogenic viruses and Hepatitis C virus.^12^ Importantly, STAT3 and suppressor of cytokine signalling‐3 (SOCS3) have also been noticed as major mediators of the outcome of Mtb infection.^13^ Phosphorylated STAT3 (pSTAT3, activated) translocates as homo‐ or heterodimers into the nucleus, where it binds to promoter sequences of specific genes or cytokines and regulates gene transcription.^11^ MicroRNAs (miRNAs), a type of non‐coding RNAs with the major role in messenger RNA (mRNA) regulation, are involved in diverse important cellular processes and in inflammation and innate immune responses.^14^, ^15^ In this paper, integrated miRNA microarray analysis, expression examination and online prediction were performed to identify the major differentially expressed miRNAs influenced by pSTAT3, with miR‐19b and miR‐1281 identified as two main subjects. Both miR‐19b and miR‐1281 have been studied to play versatile roles in many human malignancies as well as inflammatory responses.^16^, ^17^, ^18^ However, their roles in immune responses to bacterial infection remain largely unknown. Intriguingly, nuclear factor of activated T‐cells 5 (NFAT5) was predicted as a shared target mRNA of miR‐19b and miR‐1281 according to the bioinformatics analysis. Also known as tonicity‐responsive enhancer binding protein (TonEBP), NFAT5 is one of Rel homology domain (RHD) proteins and shows potentials in immune response and inflammation regulations.^19^ Taken together, this study was performed to evaluate the roles of pSTAT3 in Mtb infection‐induced lung injury and inflammation and the potential molecules involved using a mouse model with T2DM‐PTB.

## 2 METHODS AND MATERIALS

### 2.1 Ethics statement

This research was conducted with the approval of the Ethics Committee of Center for Disease Control and Prevention of Changji Hui Autonomous Prefecture. All experimental procedures were in line with the ethical guidelines for the study of experimental pain in conscious animals. Great efforts were made to minimize the usage and suffering of animals.

### 2.2 Experimental reagents

Streptozotocin (STZ) was purchased from Sigma‐Aldrich Chemical Company while nicotinamide (NA) was from Beyotime Biotechnology Co., Ltd. Enzyme‐linked immunosorbent assay (ELISA) kits of interleukin (IL)‐6 (#ab100712), IL‐1β (#ab197742) and tumour necrosis factor‐α (TNF‐α, #ab208348) were purchased from Abcam Inc. The antibody against NFAT5 (#ab6721) and horseradish peroxidase (HRP)‐labelled immunoglobulin G (IgG, #ab6721) were purchased from Abcam as well, while the antibody against pSTAT3 (#10253‐2‐AP) was acquired from Proteintech Group, Inc. The agomiR and antagomiR of miR‐19b and miR‐1281 (Sequences listed in Table 1), and the short hairpin (sh) RNA and lentiviral vector (LV) of NFAT5 were purchased from OriGene Technologies, Inc. The primer sequences for quantitative polymerase chain reaction (qPCR) including miR‐19b (#MP300180), miR‐1281 (#MP300107), miR‐378 (#MP300354), miR‐392 (#MP300488), miR‐126 (#MP300046), NFAT5 (#MP220485), U6 (#MP205832) and glyceraldehyde‐3‐phosphate dehydrogenase (GAPDH, #MP205604) were provided by Origene Technologies as well. The pSTAT3‐specific inhibitor AG‐490^20^, ^21^ (#HY‐12000) was purchased from MedChemExpress. Mtb H37Rv (25177) used for PTB induction was purchased from American Type Culture Collection (ATCC).

**Table 1:** Sequences of antagomiR or agomiR of miR‐19b/miR‐1281

	Sense	Anti‐sense
miR‐19b antagomiR	UUC UCC GAA CGU GUC ACG UTT	UGA CAC GUU CGG AGA ATT
miR‐19b agomiR	AGU UUU GCA UGG AUU UGC AC	UUU GCA UGG AUU UGC ACA UU
miR‐1281 antagomiR	AGU GAC GGU ACG GUU GUA UTT	GUC UCC UAC AGC AUC GAA UAG
miR‐1281 agomiR	UCA CUG CAU ACU GCG ACU ACC	AGC CGG UGA UUC GUC CAA GCA

### 2.3 Animal experiments

A total of 200 C57BL/6J mice (no gender limitation, 4 weeks old, 20 ± 5 g) were acquired from SLAC Laboratory Animal Co., Ltd. T2DM in mice was induced by the co‐treatment of STZ and NA. STZ was dissolved in 50 mmol/L citrate buffer, and the solution was injected into mice at 180 mg/kg for three times at a 10‐day interval through intraperitoneal injection (i.p). NA was dissolved in saline and pre‐administrated into mice 15 minutes prior to STZ injection at 60 mg/kg through i.p. The blood glucose level in mouse was determined using a blood glucose metre once a week for a total of 8 weeks. A glucose level over 250 mg/dL was considered as the successful induction of T2DM. The blood glucose level in control mice was maintained at 80‐100 mg/dL.

Mtb. H37Rv was used to induce PTB in mice. The bacteria were fed in 7H9 liquid medium till the midlogarithmic growth phase and then equally frozen at −70°C. The bacterium counting was performed on 7H10 agar plates supplemented with oil albumin dextrose catalase (OADC). For infection, the bacteria were diluted in 10 mL saline to 0.5 × 10^6^ colony‐forming units (CFU)/mL, 1 × 10^6^, 2 × 10^6^ and 1 × 10^6^ CFU/mL, respectively, and then loaded in an atomizer for aerosol exposure. In the preliminary experiments, each cage with 3 mice was exposed to aerosol with different H37Rv concentration for 15 minutes. Twenty‐four hours later, the mice were euthanized, and the animal death was confirmed by a loss of nerve reflex, blink reflex, and heartbeat, and the possibility of cardiac arrest was excluded. Then, the whole lung homogenized samples were coated on 7H10 agar plates containing OADC. The CFU in lung tissues was determined after a 22‐day incubation at 37°C with 5% CO_2_. For subsequent studies, we chose the concentration that deposited ~100 bacteria in the lung during aerosol exposure.

A month later, the pSTAT3‐specific inhibitor AG‐490 (1 mg/mL/kg), miR‐19b/1281 agomiR (5 nmol), or the shRNA or LV of NFAT5 (5 × 10^8^ PFU) was given into the mice twice every 10 days through i.p. Another month later, the mice were collected for further experiments. Ten mice in each group were used for survival test, and then the number of mice in each group was supplemented to 8. The mouse serum was collected for inflammatory cytokine detection. After mouse euthanasia, the lung tissues were collected, and the left tissues were cut into slices for histopathologic staining, while the right tissues were made into homogenate for RNA and protein expression detection.

### 2.4 Histopathologic staining

The lung tissues were fixed in 4% paraformaldehyde for 48 hours, embedded in paraffin, cut into slices (4 μm). The sections then underwent haematoxylin and eosin (HE) staining and Masson's trichrome staining. In brief, for HE staining, the tissue slices were deparaffinated and stained with haematoxylin and eosin for histological examination. For Masson's trichrome staining, the slices were treated sequentially with haematoxylin and ferric oxide, acid fuchsin, phosphomolybdic acid and acetic acid, and then mounted with neutral gum. Photographs were taken and dermal thickness was measured in the HE slices (Leica, Germany; DMI4000B). Then the histopathologic scoring was performed based on the inflammatory infiltration in lung tissues ranging from 0‐4 ^22^: (0) no inflammation, (1) a 1%‐25% infiltration area, (2) a 26%‐50% infiltration area, (3) a 51%‐75% infiltration area and (4) a 76%‐100% infiltration area. Each tissue slice was determined under a microscope with 8 fields randomly selected to evaluate the ratio of fibrous tissues. The fibrosis area was determined using an Image‐ProRPlus 6.0 Software (Media Cybernetics, MD, USA).

### 2.5 Terminal deoxynucleotidyl transferase‐mediated dUTP nick end labelling

Lung tissue slices were reacted with 20 μg/mL proteinase K (Cat. No. 1745723; Roche Ltd.) at 37°C for 15 minutes. Next, the slices were washed in phosphate buffered saline (PBS, 0.01% mol/L, pH = 7.4) and blocked at 37°C. Then, the slices were incubated with 50 μL terminal deoxynucleotidyl transferase enzyme buffer in a wet box at 37°C for an hour. Then, the slices were washed in PBS, reacted with 2% dUTP reagent for 5 minutes, washed in PBS again, and then treated with 50 μL blocking reagent to block the non‐specific binding. Next, the slices were co‐incubated with HRP‐labelled POD (Sigma‐Aldrich) at 37°C for 30 minutes. Then the slices were washed in PBS and developed using 2,4‐diaminobutyric acid (DAB), while the nuclei were counter‐stained by haematoxylin.

### 2.6 ELISA

Protein levels of IL‐6, IL‐1β and TNF‐α in serum and tissue homogenate were determined using the ELISA kits in strict accordance with the manufacturer's protocols. The measurement was performed on 96‐well plates, and the optical density (OD) value at the wavelength of 450 nm was determined using a spectrophotometer.

### 2.7 Measurement of serum lipid levels

One month after AG‐490, miR‐19b/1281 agomiR/antagomiR, or the shRNA/LV of NFAT5 treatment, the level of free fatty acid (FFA) was determined using fluorometry, while the level of triglyceride was evaluated using colorimetry on a BioVision™ assay kit (Bioptics) as per the kit's instructions.

### 2.8 Immunohistochemistry staining

The lung tissue slices were dewaxed, rehydrated, and subjected to immunohistochemical staining using a two‐step assay kit (PV‐9000; Zsbio Commerce Store, Inc.) according to the kit's instructions. The slices were co‐incubated with primary antibody pSTAT3 (1:200; Proteintech) at 4°C overnight, and then with an HRP‐labelled secondary antibody 37°C for 1 hour.

### 2.9 Reverse transcription‐qPCR

Total RNA from lung tissues was extracted using an RNApure Total RNA Fast Extraction Kit (BioTeke Corporation) and reversely transcribed into cDNA using a PromeScript RT Kit (Takara Biotechnology Ltd.) as per the kit's instructions. Relative gene expression was determined using a real‐time qPCR Kit (Gene Copoeia) on a PCR System (75000; Applied Biosystems) using the 2^‐ΔΔ^ *^C^* ^t^ method with U6 as the internal reference for miRNAs while GAPDH for mRNA.

### 2.10 Determination of differentially expressed miRNAs

The whole‐transcriptome library was prepared using a Ribo‐Zero Magnetic Gold Kit (Illumina) and a NEBNext RNA Library Preparation Kit (New England Biolabs). Extracted RNA from lung tissues was quality‐controlled and quantified on a BioAnalyzer 2100 Systerm (Kapa Biosystems). The library sequencing was performed on a HiSeq 2000 Instrument (Illumina). According to the instructions, 1 μg of total RNA was used for RNA library preparation. The fragments less than 10 nt or over 34 nt were discarded. The pure reads were compared to the miRNA precursors in the miRBase 22.1 to validate the outcomes.

### 2.11 Chromatin immunoprecipitation ‐qPCR

The binding relationships between pSTAT3 and the promoter sequence of miR‐378, miR‐19b or miR‐1281 were first predicted on JASPAR (http://jaspar.genereg.net/).^23^ Then, a chromatin immunoprecipitation (ChIP) analysis was performed using a ChIP assay kit (26157; Thermo Fisher Scientific Inc.). In brief, approximately 1 × 10^7^ cells were fixed in 1% methanol and quenched using glycine. Then, the cells were washed in PBS, soaked in 1% halt cocktail‐supplemented cold PBS and lysed by MNase and detached. Next, the mixture was subjected to ultrasonic treatment to destruct the nuclear membranes. The lysates were co‐incubated with anti‐pSTAT3 and protein G magnetic beads at 4°C overnight with normal rabbit antibody to IgG for negative control. The beads were washed 4 times, and the DNA was eluted using ChIP elution buffer, and the buffer was warm‐incubated at 65°C for 1.5 hours. Next, a DNA purification kit was used for DNA recovery, and the purified DNA was determined using qPCR. These procedures were performed in triplicate.

### 2.12 Dual‐luciferase reporter gene assay

HEK293T cells were purchased from ATCC and incubated in Roswell Park Memorial Institute‐1640 (Life Technologies) supplemented with 10% foetal bovine serum (Gibco, Thermo Fisher). The medium was refreshed every 48 hours. The 3′UTR of NFAT5 was amplified through PCR and cloned to the pGL3 vectors. HEK293T cells were seeded into 24‐well plates. When the cell confluence reached 70%‐80%, the wild type (WT) and mutant type (MT) NFAT5 3′UTR reporter vectors were constructed and co‐transfected either with miR‐19b/1281 mimic or mimic control into the HEK293T cells using the Lipofectamine 2000™ (Invitrogen). Twenty‐four hours after transfection, the relative luciferase activity was determined using a dual‐luciferase reporter kit (Promega Corporation). The firefly luciferase activity was normalized to the ranilla luciferase activity.

### 2.13 Western blot analysis

Protein level of NFAT5 was determined by Western blot analysis. In brief, total protein from lung tissues was collected. Then, 40 μg protein sample was run on 12% sodium dodecyl sulphate‐polyacrylamide gel electrophoresis and transferred onto polyvinylidene fluoride membranes. The membranes were blocked by 5% skimmed milk for 2 hours, and then incubated with the primary antibody at 4°C overnight. After PBS washes, the membranes were further incubated with HRP‐conjugated secondary antibody at room temperature for 2 hours. The membranes were visualized by the enhanced chemiluminescence reagent (EMD; Millipore), and the protein bands were analysed using the Image J 1.41 software (National Institutes of Health).

### 2.14 Statistical analysis

SPSS 21.0 was used for data analysis (IBM Corp. Armonk, NY, USA). Data were in normal distribution according to Kolmogorov‐SmiRnov test and presented as mean ± standard deviation (SD). The *t* test was used for data comparison between two groups, while one‐way or two‐way analysis of variance (ANOVA) was used among multiple groups with Tukey's multiple comparisons test used for post hoc test. The survival curve was plotted using the Kaplan‐Meier method and analysed using the log rank test. Enumeration data were compared using the Fisher's exact test. *P* was obtained from two‐tailed tests, and *P* < .05 was considered statistically significant.

## 3 RESULTS

### 3.1 Expression of pSTAT3 is increased in a mouse model with T2DM‐associated PTB

**Figure 1:**
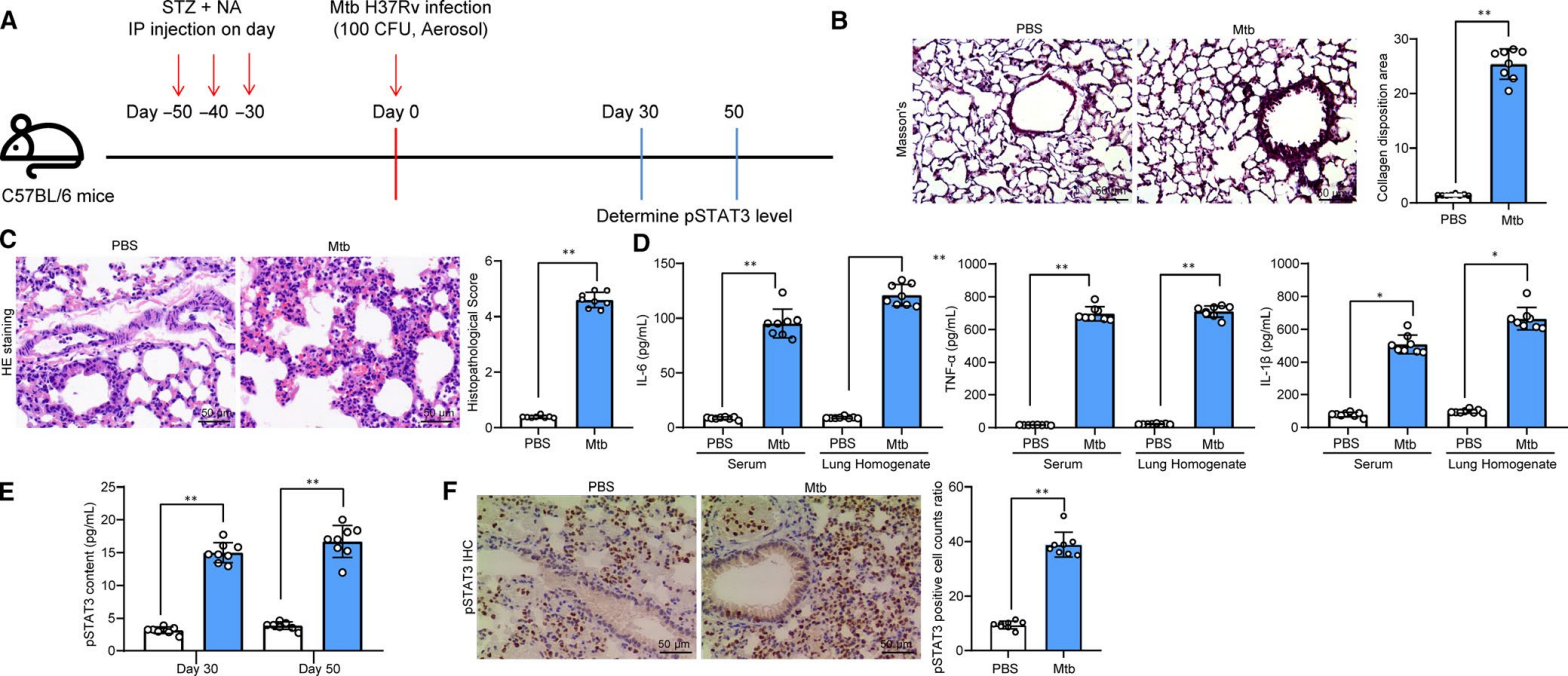
Expression of pSTAT3 is increased in mice model with T2DM‐associated PTB. A, 1 mo after diabetes induction, mice were further exposed to ~100 CFU of aerosolized Mtb; B, fibrosis in mouse lung tissues measured by Masson's trichrome staining; C, histopathologic changes in mouse lung tissues determined by HE staining; D, protein levels of inflammatory factors (IL‐6, IL‐1β and TNF‐α) in lung tissue homogenate determined using ELISA kits; E, expression level of pSTAT3 in lung tissue homogenate determined by ELISA; F, pSTAT3 expression and distribution in tissue sections evaluated by immunohistochemistry staining. Each spot in the images indicates one sample. N = 8 in each group. Three independent experiments were performed. Data were expressed as mean ± SD as the outcome of the unpaired *t* test. *P < .05, **P < .01

One month after STZ and NA co‐treatment, the T2DM mice were further infected with Mtb to acquire PTB, and a diagram for model establishment is presented in Figure 1A. Thereafter, Masson's trichrome staining was performed, and a notable increase was found in lung fibrosis in Mtb‐infected mice compared to the PBS‐treated ones (Figure 1B). In addition, the HE staining presented a large scale of inflammatory cell infiltration with disordered lung markings in the lung tissues after Mtb infection (Figure 1C). According to the ELISA results, the production of pro‐inflammatory cytokines including IL‐6, IL‐1β and TNF‐α in mouse lung tissues was notably increased following Mtb infection (Figure 1D). On the 50th day after Mtb infection, pSTAT3 expression in mouse lung homogenate, according to the ELISA kit detection again, was notably enhanced (Figure 1E). Likewise, the immunohistochemical staining identified a high expression level of pSTAT3 in mouse lung tissues, while was mainly in nuclei (Figure 1F).

### 3.2 Inhibition of pSTAT3 prolongs the lifetime of mice with T2DM‐associated PTB

**Figure 2:**
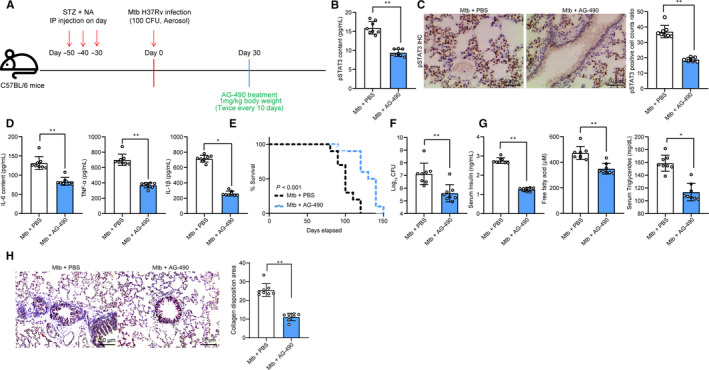
Inhibition of pSTAT3 prolongs the lifetime of mice with T2DM‐associated PTB. A, 1 mo after diabetes induction, mice were exposed to ~100 CFU of aerosolized Mtb; another 30 d later, each mouse was further given AG‐490 (1 mg/kg) twice every 10 d through i.p for a total of 30 d; B‐C, pSTAT3 expression in mouse lung tissues determined by ELISA (B) and immunohistochemistry staining (C); D, protein levels of TNF‐α, IL‐6 and IL‐1β in lung tissue homogenate evaluated using ELISA kits; E, survival time of mouse treated with AG‐490 (verses PBS) after Mtb infection (n = 10); F, Mtb content in mouse lung tissues; G, level of FFA by fluorometry while the level of triglyceride by colorimetry; H, fibrosis in mouse lung tissues determined by Masson's trichrome staining. Each spot in the images indicates one sample. N = 8 in each group. Three independent experiments were performed. Data were expressed as mean ± SD as the outcome of the unpaired *t* test. *P < .05, **P < .01

To further identify the roles of pSTAT3 in lung injury in mice with T2DM‐associated PTB, 1 month after Mtb infection, each mouse was given AG‐490 (1 mg/kg) twice every 10 days through i.p for a total of 30 days (Figure 2A). Then, the ELISA and immunohistochemical staining results identified that the pSTAT3 expression was notably decreased following AG‐490 administration (Figure 2B‐C). ELISA results also identified that the levels of TNF‐α, IL‐6 and IL‐1β in lung tissue homogenate were decreased when pSTAT3 was reduced (Figure 2D). In addition, we found that the median lifetime of mice with AG‐490 treatment was 8.4 months (median survival days 252.7), while that of the control mice (PBS treatment) was 6.7 months (median survival days 204.3) (Figure 2E). Importantly, it was found that AG‐490 treatment led to a notable decline in Mtb concentration in mouse lung tissues (Figure 2F). Moreover, we also found that the levels of FFA and triglyceride in serum were decreased after AG‐490 treatment (Figure 2G). In addition, the Masson's trichrome staining suggested that when pSTAT3 was suppressed by AG‐490, the collagen deposition in mouse lung tissues was notably decreased, namely the tissue fibrosis was reduced (Figure 2H).

### 3.3 pSTAT3 transcriptionally suppresses miR‐19b/1281 to up‐regulate NFAT5 expression

**Figure 3:**
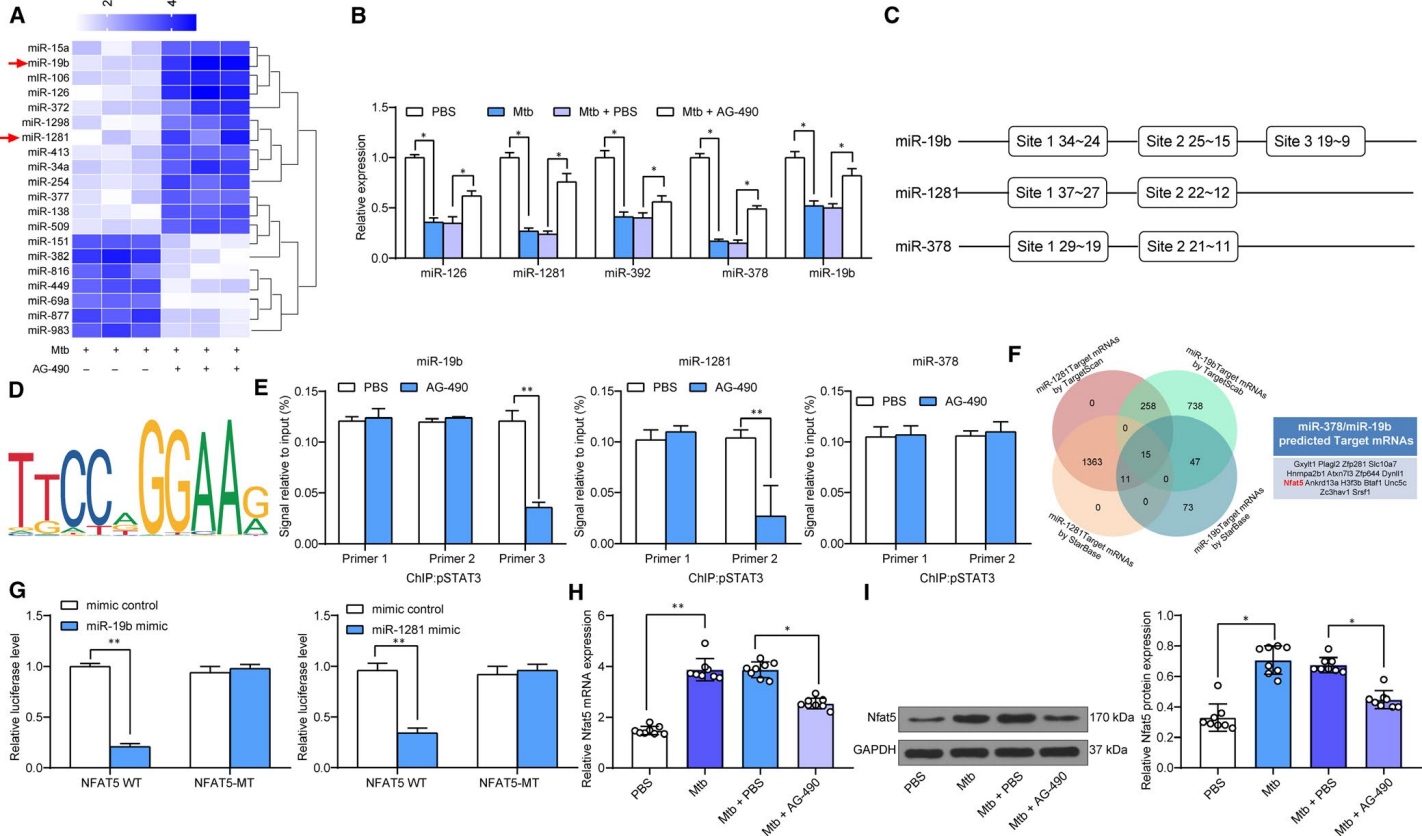
pSTAT3 transcriptionally suppresses miR‐19b/1281 to up‐regulate NFAT5 expression. A, a heatmap for top 20 differentially expressed miRNAs in mouse lung tissues following AG‐490 treatment determined by a miRNA transcriptome analysis; B, miR‐126, miR‐19b, miR‐392, miR‐1281 and miR‐378 expression in lung tissues evaluated using RT‐qPCR; C, binding sites between pSTAT3 and the promoter sequences of miR‐19b, miR‐1281 and miR‐378 predicted on JASPAR System; D, conservative binding sequence of pSTAT3; E, binding relationship between pSTAT3 and miR‐19b, miR‐1281 or miR‐378 validated by ChIP‐qPCR; F, shared downstream target mRNAs of miR‐19b and miR‐1281 predicted on StarBase and TargetScan; G, binding relationship between miR‐19b/1281 and NFAT5 validated by dual‐luciferase reporter gene assays; H‐I, NFAT5 expression in mouse lung tissues determined by RT‐qPCR (H) and Western blot analysis (I), respectively. Three independent experiments were performed. N = 8 in each group. Data were expressed as mean ± SD. In panels H and I, data were analysed using one‐way ANOVA, while data in panels B, E and G were determined by two‐way ANOVA, and Tukey's multiple comparison for post hoc test after ANOVA. *P < .05, **P < .01

To identify the potentially involved molecules, a miRNA transcriptome analysis was performed to identified the differentially expressed miRNAs in mouse lung tissues following AG‐490 treatment. A total of 341 miRNAs were significantly changed under the |Log_2_FC > 2| and *P* < .05 criteria, and a heat map for top 20 differentially expressed miRNAs is presented in Figure 3A. We then measured miR‐126, miR‐19b, miR‐392, miR‐1281 and miR‐378 expression in lung tissues using reverse transcription‐qPCR (RT‐Qpcr), and found the expression of these miRNAs was initially decreased after Mtb infection but then increased following further AG‐490 treatment (Figure 3B). Since pSTAT3 can transcriptionally regulate gene expression, we then predicted the binding relationships between pSTAT3 and the promoter sequences of the 3 mostly changed miRNAs miR‐19b, miR‐1281 and miR‐19a on JASPAR bio‐information system (Figure 3C‐D). The ChIP‐qPCR was performed and identified that pSTAT3 could suppress miR‐19b and miR‐1281 transcription but excluding miR‐19a (Figure 3E). After that, we further searched for the shared target mRNAs of miR‐19b and miR‐1281 on StarBase (http://starbase.sysu.edu.cn/) (https://doi.org/10.1093/nar/gkt1248) and TargetScan (http://www.targetscan.org/vert_72/) (https://doi.org/10.7554/eLife.05005.001) with 15 mRNAs identified (Figure 3F). Dual‐luciferase reporter gene assays were performed to validate the binding relationships between miR‐19b/1281 and NFAT5, and the results showed that miR‐19b/1281 could stably bind to NFAT5 (Figure 3G). Thereafter, RT‐qPCR and Western blot analysis was further performed and identified an increase in NFAT5 expression after Mtb infection while a decline following further AG‐490 administration (Figure 3H‐I).

### 3.4 miR‐19b/1281 agomiR or sh‐NFAT5 reduces inflammation in lung tissues and lung epithelial cell apoptosis in T2DM‐PTB mice

**Figure 4:**
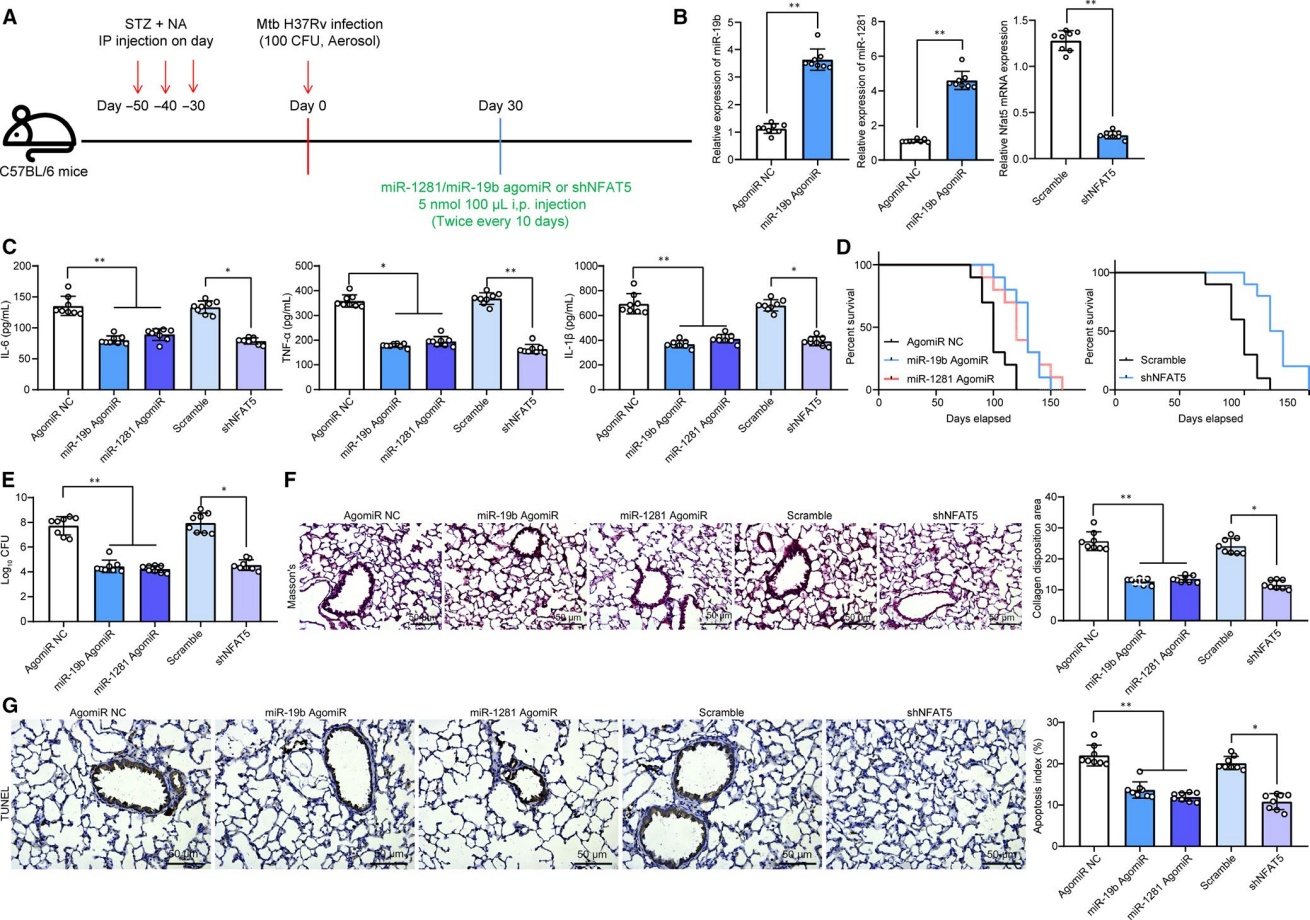
miR‐19b/1281 agomiR or sh‐NFAT5 reduces inflammation in lung tissues and lung epithelial cells in T2DM‐PTB mice. A, 1 mo after diabetes induction, mice were exposed to ~100 CFU of aerosolized Mtb; another 30 d later, each mouse was further given miR‐19b/1281 agomiR, or the shRNA of NFAT5 (5 nmol/100 μL) twice every 10 d through i.p for a total of 30 d; B, expression of miR‐19b, miR‐1281 and NFAT5 mRNA in lung tissue homogenate determined by RT‐qPCR; C, protein levels of TNF‐α, IL‐6 and IL‐1β in lung tissues determined by ELISA kits; D, survival time of mice after Mtb infection (n = 10); E, Mtb content in mouse lung tissues; F, lung fibrosis detected using Masson's trichrome staining; G, apoptosis in lung tissues determined by dUTP nick end labelling (TUNEL). Each spot in the images indicates one sample. N = 8 in each group. Three independent experiments were performed. Data were expressed as mean ± SD. In panel B, data were analysed using the unpaired *t* test, while data in panels C, E, F and G were analysed by one‐way ANOVA and Tukey's multiple comparison test. *P < .05, **P < .01

To further confirm the effects of above molecules in lung injury in model mice, 1 month after Mtb infection, miR‐19b/1281 agomiR, or the shRNA of NFAT5 (5 nmol/100 μL) was given into the mice twice every 10 days through i.p. (Figure 4A). Thereafter, the miR‐19b/1281 expression was increased and NFAT5 expression was decreased according to RT‐qPCR results (Figure 4B). Next, we found that overexpression of miR‐19b or miR‐1281, or down‐regulation of NFAT5 led to a decline in the levels of TNF‐α, IL‐6 and IL‐1β in lung tissues (Figure 4C), and the lifetime of model mice was prolonged as well (Figure 4D). In addition, we also found that the CFU of Mtb in mouse lung was notably reduced after miR‐19b/1281 up‐regulation or NFAT5 down‐regulation (Figure 4E). In the meantime, the fibrosis in lung tissues was decreased following miR‐19b/1281 agomiR, or NFAT5 shRNA administration (Figure 4F). The apoptosis of lung epithelial cells was inhibited as well (Figure 4G).

### 3.5 Lv‐NFAT5 partially blocks the effects of miR‐19b/miR‐1281 agomiR

**Figure 5:**
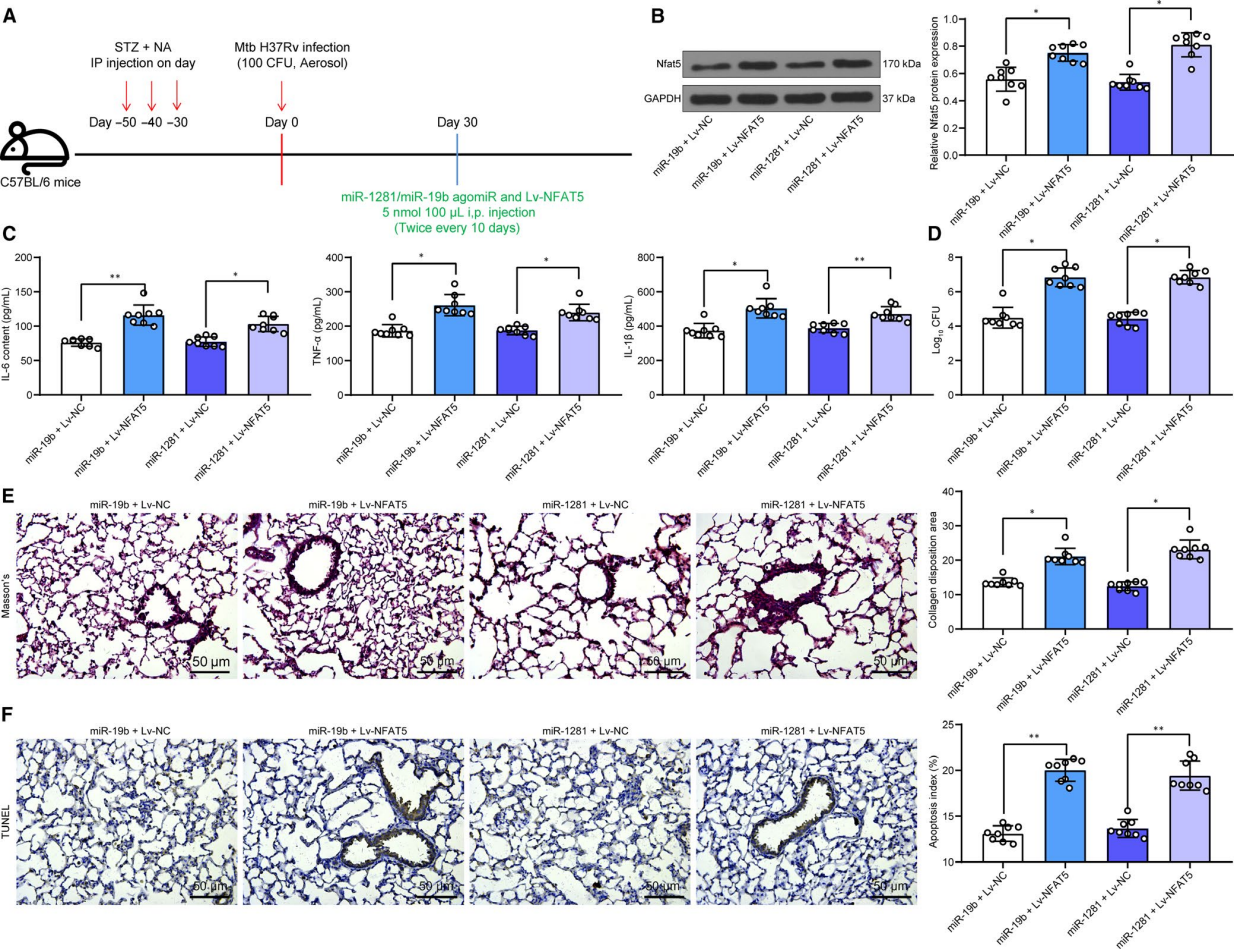
Lv‐NFAT5 partially blocks the effects of miR‐19b/miR‐1281 agomiR. A, 1 mo after diabetes induction, mice were exposed to ~100 CFU of aerosolized Mtb; another 30 d later, each mouse was further given miR‐19b/1281 agomiR, or the LV overexpressing NFAT5 (5 nmol/100 μL) twice every 10 d through i.p for a total of 30 d; B, protein level of NFAT5 in mouse lung tissues determined by Western blot analysis; C, secretion of pro‐inflammatory factors in mouse lung tissue homogenate determined by ELISA kits; D, Mtb content in lung tissues; E, lung fibrosis in mouse lung tissues determined by Masson's trichrome staining; F, apoptosis in lung tissues determined by TUNEL. Each spot in the images indicates one sample. N = 8 in each group Three independent experiments were performed. Data were expressed as mean ± SD compared by one‐way ANOVA and Tukey's multiple comparison test. *P < .05, **P < .01

Following the findings above, we further co‐transfected LV‐NFAT5 (overexpressing NFAT5) and miR‐19b/miR‐1281 agomiR in mouse 30 days after Mtb infection (Figure 5A). Then, the NFAT5 expression in mouse lung tissue homogenate was increased according to the Western blot analysis (Figure 5B). Then, it was found that overexpression of NFAT5 promoted the inflammatory reactions, the CFU of Mtb, the lung fibrosis and lung epithelial cell apoptosis that were inhibited by miR‐19b/miR‐1281 agomiR (Figure 5C‐F).

### 3.6 miR‐19b/1281 antagomiR inhibits the functions of AG‐490 in mice

**Figure 6:**
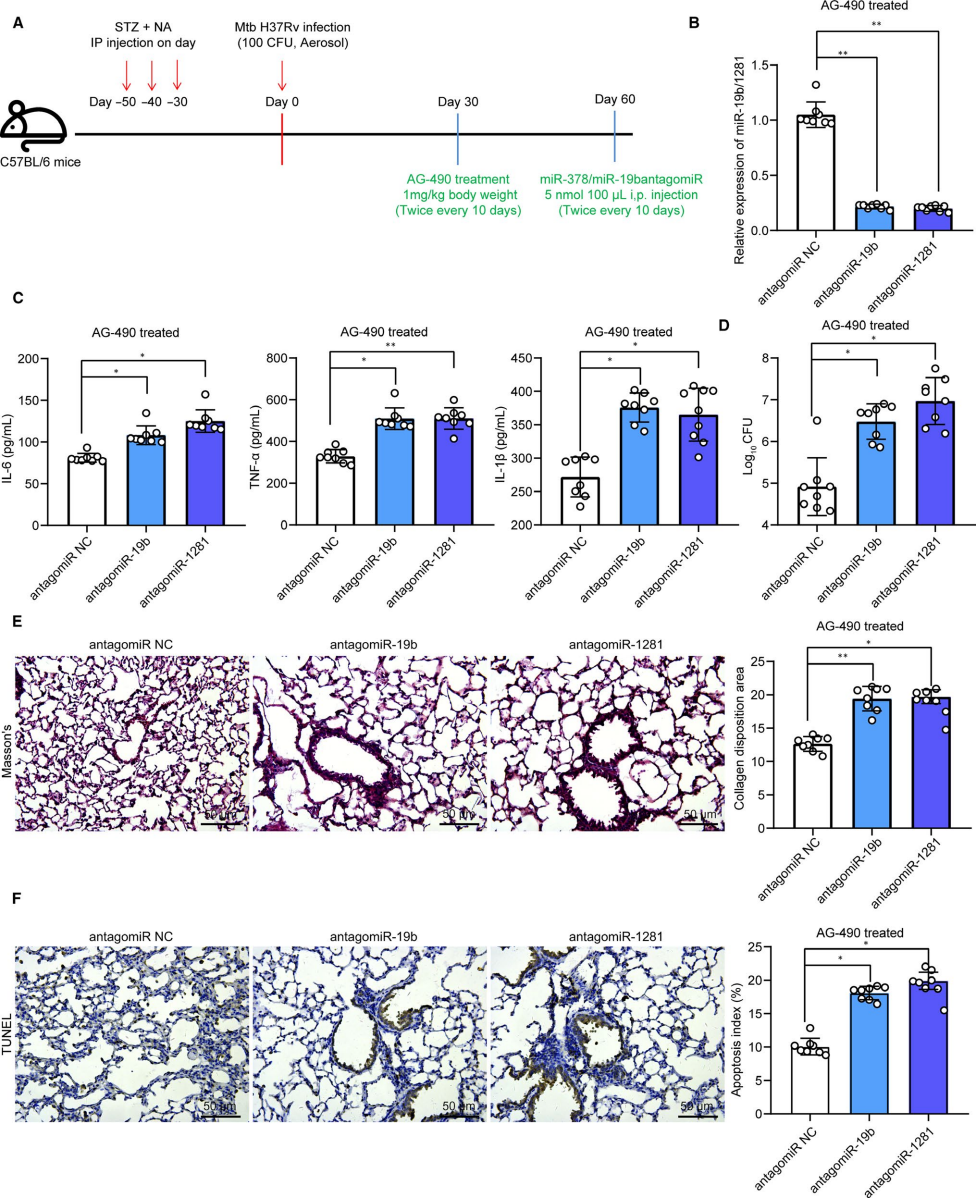
miR‐19b/1281 antagomiR inhibits the functions of AG‐490 in mice. A, 1 mo after ~100 CFU of aerosolized Mtb infection, mice were given with AG‐490 (1 mg/kg) through i.p twice every 10 d through i.p for a total of 30 d, another 30 d later, each mouse was further given miR‐19b/1281 agomiR (5 nmol/100 μL); B, expression of miR‐19b/1281 in mouse lung tissues determined by Western blot analysis; C, secretion of pro‐inflammatory factors in mouse lung tissue homogenate determined by ELISA kits; D, Mtb content in lung tissues; E, lung fibrosis in mouse lung tissues determined by Masson's trichrome staining; F, apoptosis in lung tissues determined by TUNEL. Each spot in the images indicates one sample. N = 8 in each group. Three independent experiments were performed. Data were expressed as mean ± SD compared by one‐way ANOVA and Tukey's multiple comparison test. *P < .05, **P < .01

To further validate the involvement of miR‐1281/19b in AG‐490‐mediated events, 30 days after AG‐490 treatment, cells were further treated with miR‐19b/1281 antagomiR (5 nmol/100 μL) twice every 10 days through i.p for a total of 30 days (Figure 6A). Then, it was found that the miR‐19b/1281 expression in lung tissues was declined (Figure 6B). ELISA results found that the IL‐6, IL‐1β and TNF‐α levels in lung tissues were increased following miR‐19b/1281 down‐regulation (Figure 6C). Accordingly, the Mtb content, lung fibrosis and apoptosis of lung epithelial cells inhibited by AG‐490 were aggravated when miR‐19b/1281 was inhibited (Figure 6D‐F).

### 3.7 Overexpression of NFAT5 blocks the roles of AG‐490 in mice

**Figure 7:**
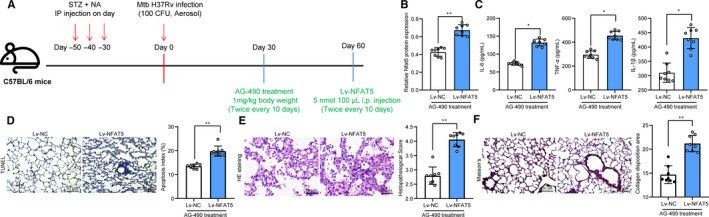
Overexpression of NFAT5 blocks the role of AG‐490 in mice. A, 1 mo after ~100 CFU of aerosolized Mtb infection, mice were given with AG‐490 (1 mg/kg) through i.p twice every 10 d through i.p for a total of 30 d, another 30 d later, each mouse was further given LV overexpressing NFAT5 (5 nmol/100 μL); B, protein level of NFAT5 in mouse lung tissues determined by Western blot analysis; C, secretion of IL‐6, IL‐1β and TNF‐α in mouse lung tissue homogenate determined by ELISA kits; D, number of apoptotic epithelial cells determined by TUNEL; E, histopathologic changes in mouse lung tissues observed through HE staining F, lung fibrosis in mouse lung tissues determined by Masson's trichrome staining. Each spot in the images indicates one sample. N = 8 in each group. Three independent experiments were performed. Data were expressed as mean ± SD compared by the unpaired *t* test. *P < .05, **P < .01

In addition, T2DM‐PTB mice treated with AG‐490 were further administrated with LV‐NFAT5 (Figure 7A). The Western blot analysis identified an increased level of NFAT5 in mouse tissue homogenate (Figure 7B). Reasonably, it was found that the secretion of inflammatory cytokines and Mtb content as well as epithelial cell apoptosis in lung tissues inhibited by AG‐490 was promoted after NFAT5 overexpression (Figure 7C‐D). In addition, the HE staining found that the inflammatory cell infiltration in lung tissues was increased and the Masson's trichrome staining identified increased fibrosis in lung tissues (Figure 7E‐F).

## 4 DISCUSSION

Given to the increasing incidence of Mtb reactivation and the especially high recurrence and mortality rate in TB patients associated with T2DB, the control of Mtb infection and TB‐related death remains a great challenge. Importantly, STAT3 activation has been noted to be crucial for inflammatory diseases and in bacterial infection as well as the outcome of Mtb infection,^13^, ^24^ but the potentially involved molecules remain complicated and elusive. Here, our study identified that pSTAT3 aggravated lung injury in a T2DM‐associated PTB murine model through transcriptionally suppressing miR‐19b and miR‐1281 which up‐regulated NFAT5 expression.

Inflammation is a typical event of chronic diseases including PTB.^25^ The successful establishment of a mouse model with T2DM‐associated PTB was confirmed as inflammatory cell infiltration and increased pulmonary fibrosis, as well as increased production of pro‐inflammatory factors including TNF‐α, IL‐6 and IL‐1β in mouse lung tissues. Elevated expression of TNF‐α and IL‐6 has been witnessed in patients with PTB or with T2DM‐PTB vs that in healthy individuals.^2^, ^26^ We then found that pSTAT3 expression was notably increased 30 days after Mtb infection in model mice. Next, artificial inhibition of pSTAT3 reduced the inflammatory response, fibrosis in lung tissues, decreased the levels of FFA and triglyceride in mouse serum and the CFU of Mtb in lung, and accordingly, prolonged the survival time of T2DB‐PTB mice. STAT3 plays key functions in bacterial infection and inflammatory diseases, whose activation renders host‐bacterial interactions, mainly by controlling bacterial growth and reducing apoptosis.^11^ STAT3 was linked to an increased pro‐inflammatory response, and its phosphorylation has been noted to hold accountable for excessive secretion of TNF‐α in neuroinflammation.^27^ Intriguingly, IL‐6 receptor is crucial for STAT3 phosphorylation ^13^ while TNF‐α was capable of further promoting the IL‐6 induced phosphorylation.^28^ In addition, STAT3 was found to induce M2 polarization of macrophages which can enhance the survival of Mtb.^26^ This may attribute to decreased production in nitric oxide which shows strong anti‐bacterial strategy and limits the proliferation of intracellular pathogens including Mtb.^29^ This pro‐survival role of STAT3 in Mtb was further evidenced by reduced CFU of Mtb following STAT3 silencing.^29^ Likewise, STAT3 activation has also been documented to increase the risk of successful Mtb infection in mice.^24^

As a transcription factor, pSTAT3 is capable of regulating gene expression transcriptionally. After identification of the nuclear abundance of the pSTAT3 sub‐cellular localization, we determined the potential miRNAs regulated by pSTAT3 through integrated miRNA microarray analysis, RT‐qPCR, online prediction and ChIP‐qPCR assays. Consequently, miR‐19b and miR‐1281 found as two major miRNAs that were up‐regulated following AG‐490 treatment. miRNAs play versatile roles in innate immune response and bacterial infection in lung.^30^, ^31^ For instance, miR‐27b was found to suppress the secretion of pro‐inflammatory factors and the nuclear factor‐kappa B (NF‐κB) signalling pathway to avoid an excessive inflammation during Mtb infection.^32^ Likewise, miR‐20b was suggested to suppress Mtb‐induced inflammation in lung of mice through directly binding to NLRP3 inflammasome.^33^ In terms of miR‐19, a recent study has focused on its role in immune and inflammatory responses in human cancers.^34^ In addition, protective roles of miR‐19b‐3p in patients with sepsis have been identified as well by relieving the levels of pro‐inflammatory factors IL‐6 and TNF‐α.^35^ MiR‐19b‐3p was also reported to attenuate IL‐1β‐induced inflammatory injury in chondrocytes through targeting GRK6.^16^ As for miR‐1281, it has been identified as a tumour suppressor in many malignancies such as glioma,^17^ osteosarcoma ^36^ and gastric cancer.^37^ Interestingly, it also presented protective functions in macrophages against Mtb infection by reducing the programmed necrosis and apoptosis.^38^ Importantly, in this paper, our study found that up‐regulation of miR‐19b or miR‐1218 led to a decline in inflammatory cytokine production, inflammatory response, fibrosis and normal epithelial cell apoptosis in mouse lung tissues. Silencing of these miRNAs blocked the protective roles of AG‐490 against Mtb infection in mice, indicating the involvement of miR‐19b or miR‐1218 in Mtb containment following pSTAT3 down‐regulation.

Based on the findings above, we further identified NFAT5 as a targeting mRNA of both miR‐19b and miR‐1281 through online prediction and dual‐luciferase reporter genes assays. NFAT5 has been noted to play crucial functions in immune and inflammatory response regulation.^19^, ^39^ Here, the evidence that NFAT5 expression was increased following Mtb infection but reduced after AG‐490 administration suggested the potential involvement of NFAT5 in Mtb infection. Thereafter, we found artificial silencing of NFAT5 reduced inflammatory response, fibrosis and normal epithelial cell apoptosis in mouse lung tissues. Similarly, a previous study also found that both gene and protein expression of NFAT5 was induced by Mtb infection, while NFAT5 down‐regulation led to a decline in Mtb‐stimulated HIV‐1 replication in co‐infected macrophages by interacting with the toll‐like receptor pathway.^40^ Additionally, up‐regulation of NFAT5 blocked the protective roles of miR‐19b/miR‐1281 agomiR in this study, which further evidenced its implication in miR‐19b/miR‐1218‐mediated events.

To sum up, our present study suggested that pSTAT3 was at least partially responsible for Mtb‐induced inflammation and lung injury and Mtb survival in a mouse model with T2DM‐associated PTB. Silencing of pSTAT3 reduced the symptoms and inflammation as well as Mtb survival through the up‐regulation of miR‐19b/miR‐1281 which suppressed NFAT5 expression. This study may offer novel insights into T2DM‐PTB treatment. In addition, considering the possibility that AG‐490 might affect other pathways such as JAK, EGFR and STAT1/5, and we would like to discuss the potential involvement of other target pathways in the protective events mediated by AG‐490 in our future studies.

## CONFLICT OF INTEREST

The authors declare no potential conflicts of interest.

## AUTHOR CONTRIBUTIONS

**Xianhua Wang:** Conceptualization (lead); Data curation (lead); Formal analysis (lead); Methodology (lead); Writing‐review & editing (lead). **Yuefu Lin:** Formal analysis (equal); Funding acquisition (equal); Project administration (equal); Resources (equal); Software (equal); Supervision (equal). **Ying Liang:** Formal analysis (equal); Funding acquisition (equal); Investigation (equal); Methodology (equal); Visualization (equal); Writing‐original draft (equal). **Yang Ye:** Data curation (equal); Formal analysis (equal); Resources (equal); Software (equal); Supervision (equal); Validation (equal); Visualization (equal). **Dong Wang:** Investigation (equal); Methodology (equal); Project administration (equal); Software (equal); Writing‐review & editing (equal). **Aer Tai:** Formal analysis (equal); Software (equal); Visualization (equal); Writing‐original draft (equal). **Shuimiao Wu:** Investigation (equal); Methodology (equal); Software (equal); Validation (equal). **Jian Pan:** Supervision (equal); Validation (equal); Visualization (equal); Writing‐original draft (equal); Writing‐review & editing (equal).

## Data Availability

All the data generated or analysed during this study are included in this published article.
